# A Rare Case of Hemangioendothelioma of Upper Thigh

**DOI:** 10.7759/cureus.10642

**Published:** 2020-09-25

**Authors:** Kapil Baliga, Sriramulu G, Nirupama Kasturi

**Affiliations:** 1 Surgery, Indira Gandhi Medical College and Research Institute, Puducherry, IND; 2 Surgery, Indira Gandhi Government General Hospital and Research Institute, Puducherry, IND; 3 Ophthalmology, Jawaharlal Institute of Postgraduate Medical Education and Research, Puducherry, IND

**Keywords:** hemangioendothelioma, vascular tumor, angiogram

## Abstract

Hemangioendothelioma is a rare tumor arising from the vascular endothelium of soft tissue, skin, bone, and viscera. The disease has an indolent course, with the potential for recurrence, and is often associated with multi-organ involvement. We present a rare case of subcutaneous tumor in the thigh, without systemic involvement in a middle-aged woman. The tumor was completely excised after ligation of the feeding vessels, and the histopathologic diagnosis was consistent with hemangioendothelioma. Regular follow-up is suggested due to the potential for recurrence.

## Introduction

Hemangioendothelioma is a rare vascular tumor arising from the vascular endothelium of soft tissue, skin, bone, and viscera. It is considered a neoplasm of intermediate malignant potential. Soft tissue hemangioendotheliomas affect the extremities in two-thirds of cases, followed by head and neck region, trunk, mediastinum, and other sites. The lesions can be multicentric, and more than 50% of the cases develop around or from a large vein.

## Case presentation

**Figure 1:**
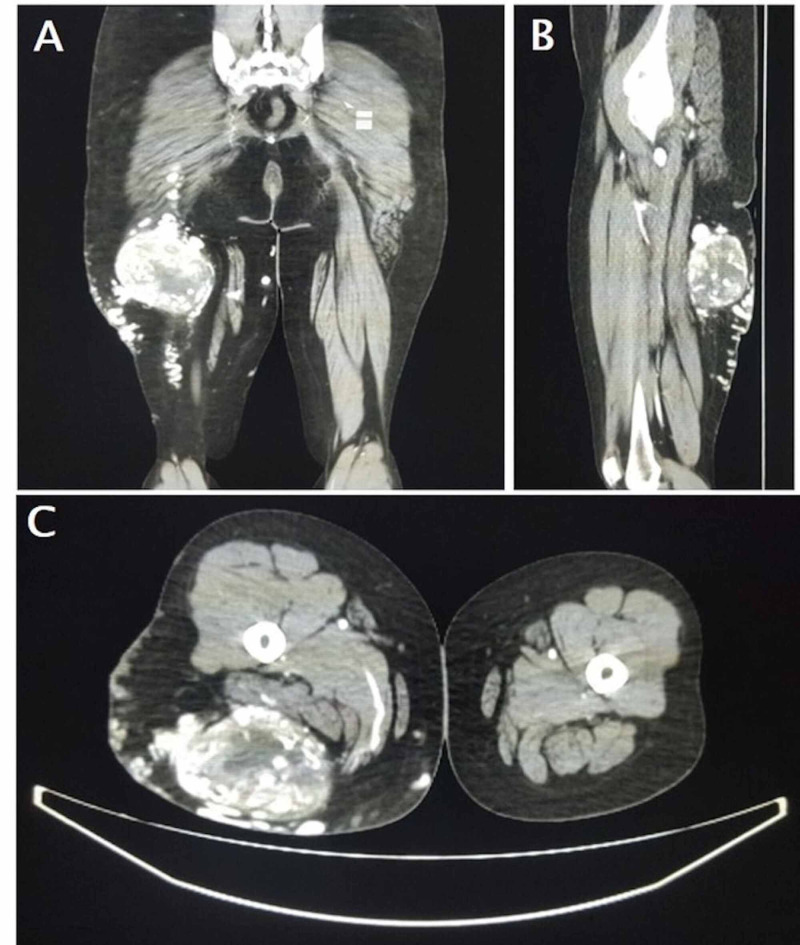
The coronal, axial, and sagittal CT scan images demonstrating the soft tissue tumor in the deep subcutaneous plane of upper thigh with no sign of bony erosion.

**Figure 2:**
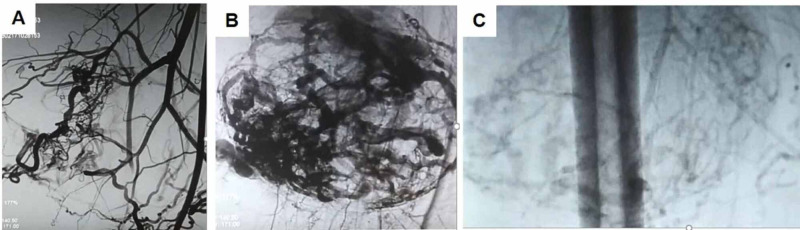
Digital subtraction angiogram showing A) the feeding vessels supplied by the right deep femoral (profunda femoris) artery B) tumor blush C) marked reduction in the feeding vessels to tumor.

**Figure 3:**
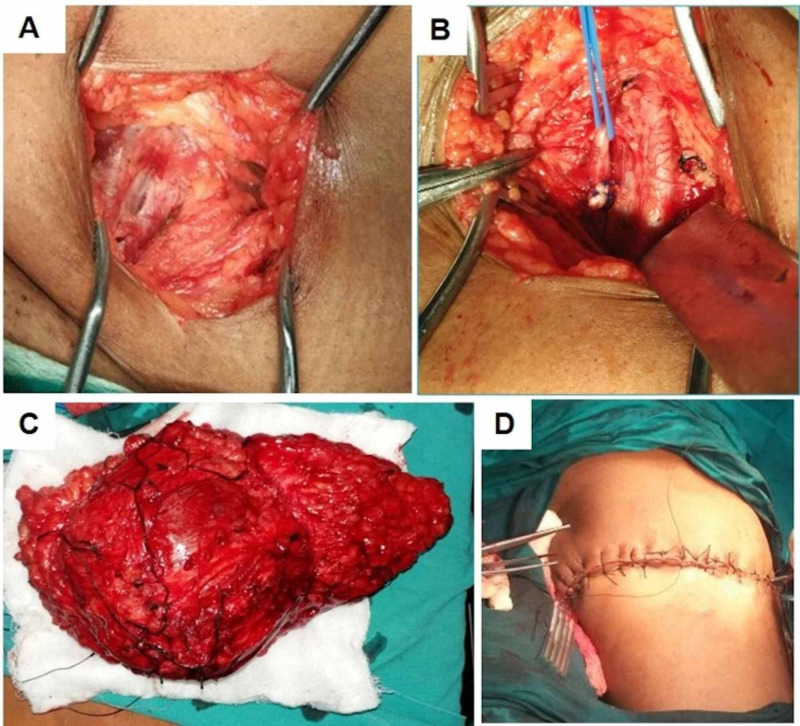
A-B) Intra-operative photograph showing ligation of the profunda femoris artery and subsequently C-D) the resected tumor.

A 38-year-old woman presented with a swelling on the back of her right thigh, which gradually increased in size for six months. It was first noticed 15 years ago with a smooth skin surface but now presenting with a few tortuosities over the swelling. It was associated with a dull aching pain with no diurnal variations, no history of any trauma or ulcerations. On examination, a 15 × 15 cm swelling was seen on the inner and posterior aspects of the thigh with an irregular surface. Dilated tortuous vessels were seen on the surface with no visible pulsations. The skin over swelling was normal. The swelling was well defined and firm in consistency, with no obvious thrill. The swelling was superficial to the muscles, and the skin was pinchable. There was no regional lymphadenopathy. Proximal and distal pulses were palpable. She underwent a CT angiogram that revealed a brightly enhancing vascular tumor occupying the posteromedial aspect of the right upper thigh in the deep subcutaneous plane with the muscles displaced away from the skin (Figure 1). A digital subtraction angiogram was also performed that revealed the vascular tumor to be mainly supplied by the right deep femoral (profunda femoris) artery, after the lateral circumflex branch has been given off (Figure 2). Therefore, based on the imaging, the patient underwent an anterior approach selective double ligation of the right profunda femoris artery (Figure 3). After three weeks, the swelling reduced by 30% and the pain reduced. A repeat angiogram showed a marked reduction in the feeding vessels to the tumor. Therefore, the patient was taken up for wide excision of the tumor. On follow-up, the patient was afebrile and ambulant, and the sutures were removed on the 10th postoperative day. Histopathology of the excised mass confirmed the diagnosis of a hemangioendothelioma. There is no sign of tumor recurrence or metastatic spread at the end of three-year follow-up.

## Discussion

Hemangioendothelioma is an uncommon, well-differentiated endothelial vascular neoplasm of low-grade malignancy belonging to the intermediate variant of the family of vascular tumors where its behavior lies between entirely benign hemangiomas and highly malignant angiosarcomas [1]. It is considered to arise from a vein [2]. It presents frequently during the second and third decades of life, but rarely occurs during childhood. It commonly arises in the liver, lung, bone, and skin, and hemangioendotheliomas in the thigh are extremely rare [3-6]. The symptoms may be variable from asymptomatic to pain. The clinical presentation is related to the location of the tumor. Hemangioendotheliomas involving the skin and soft tissues can be classified histopathologically into papillary intralymphatic angioendothelioma, retiform hemangioendothelioma, kaposiform hemangioendothelioma, epithelioid hemangioendothelioma, pseudomyogenic hemangioendothelioma (also known as epithelioid sarcoma-like hemangioendothelioma), and composite hemangioendothelioma [7]. Tumor cells are of round or polygonal endothelial cells with abundant eosinophilic cytoplasm arranged in small nests or cords. The cells often have intracytoplasmic vacuoles representing small vascular lumina is characteristic. High-risk features include nuclear atypia, increased mitotic activity, and necrosis. Immunohistochemistry may show positivity for CD31, CD34, and vimentin [8,9]. Most of these tumors are low grade; however, large lesions and those located in deep soft tissues seem to have a more aggressive biological behavior. Treatment depends on the grading, the location, and the spread of the tumor. Ligation or coiling of feeding vessels enables complete resection, with minimal blood loss. Wide resection is recommended for its curative effect. Radiotherapy can be tried for large unresectable tumors or with metastatic spread. Chemotherapy has been met with a low success rate. The mortality rates from the disease in the lung and liver are higher compared with a tumor arising in the soft tissues [10].

## Conclusions

Hemangioepithelioma of the thigh region is a rare vascular tumor, which has good prognosis if detected early and managed surgically. Larger and deeply located tumors with high mitotic activity are associated with metastasis and lower survival rates. 
